# EQ-5D-3L and quality of life in total knee arthroplasty (TKA) patients: beyond the index scores

**DOI:** 10.1186/s41687-022-00497-0

**Published:** 2022-08-30

**Authors:** David William Parkin, Bronwyn Do Rego, Rhonda Shaw

**Affiliations:** 1 City, University of London, Northampton Square, London, EC1V 0HB UK; 2 Office of Health Economics, London, UK; 3 Present Address: London, UK; 4 Present Address: Amsterdam, The Netherlands

**Keywords:** Patient reported outcome measures, EQ-5D, Total knee arthroplasty, Exploratory data analysis

## Abstract

**Background:**

Many patient reported outcome measures (PROMs) generate single number index scores that summarise responses to different questions within a questionnaire. Although these are valuable as unambiguous indicators of ill-health and changes in health, further information can be gained from examining the questionnaire responses themselves. This has additional importance since the patients’ reports are those responses and not the index scores. This paper reanalyses data from two prospective multicentre clinical studies assessing patients’ quality of life before and after total knee arthroplasty (TKA) using the EQ-5D-3L. Patients who completed EQ-5D-3L questionnaires at 3 time periods were included from 4 countries (USA, UK, Australia, New Zealand) operated on by the same surgeons using two different knee replacement systems. Descriptive analyses included levels within EQ-5D dimensions and profiles (combinations of dimensions and levels) at baseline and follow-up, including changes over time and differences between implants. Regression analysis quantified the relationship between the profiles and the EQ VAS.

**Results:**

Problems with mobility, usual activities and pain & discomfort, in that order, were most frequent for pre-operative patients. There were large improvements in every dimension after surgery, but the time that this was observed differed: for mobility, self-care and anxiety & depression, most of the improvement occurred during the first 10 months; for usual activities and pain & discomfort, over 11–22 months. Analysing profiles, 85% of patients experienced an unequivocal improvement, 5.9% had no change, 3.5% worsened and 5.5% a mixed change at 11–22 months follow-up. Anxiety & depression had the greatest impact on EQ VAS scores; while mobility and usual activities were also important; usual activities was particularly important post-surgery.

**Conclusions:**

The value of analysing patients’ responses to PROMs questionnaires without using summary scores was confirmed. The results add further detail to existing knowledge about the health problems that TKA patients experience before surgery, improvements after surgery, residual problems that some have, and the time at which improvements are experienced. This was a small study, but its methods may be easily replicated in other studies that use the EQ-5D-3L. It may also be possible to adapt them for other PROMs.

**Supplementary Information:**

The online version contains supplementary material available at 10.1186/s41687-022-00497-0.

## Introduction

Many health status and quality of life measurement instruments are designed to generate index numbers that summarise responses to different questions within a questionnaire. As patient reported outcome measures (PROMs), these index numbers are valuable as unambiguous indicators of ill-health, and changes in health, within the dimensions covered by a validated instrument. These can be used to assess patients’ clinical need and determine clinical- and cost-effectiveness of health care [1].

Broadly, index numbers are generated by assigning scores to the responses that patients provide to the questions asked within the instrument and summing them. There are variations on this; for example, some instruments divide questions into categories or ‘domains’ and calculate separate scores for them; some apply ‘weights’ to these domain scores to calculate an overall index. Instruments of this kind may be validated by correlating the resulting scores with other health state measures and with expert opinions.

It is common practice to analyse and report only the index numbers and, in effect, to regard the questionnaire responses only as contributors to calculation of the scores. However, further information can be gained from examining the responses themselves, which has additional importance since the patient reported aspect of PROMs consists of the questionnaire responses and not the index scores. The explicit or implicit weights placed on different responses derive from the instrument’s developer rather than those completing the questionnaire.

It is a simple matter to report, for example, frequency counts of responses to different questions. Of more interest is whether it is possible to gain further information by examining combinations of responses. In the extreme, is it possible to obtain an overall view of patients’ health or health-related quality of life (HRQOL) using only the responses without calculating an index score? For many PROMs, their complexity means that it is difficult to analyse the detailed information from responses in this way. For example, the widely used Oxford Hip Score and Oxford Knee Score [2] each implicitly define over 244 million unique health states. However, the EQ-5D-3L [3, 4] is sufficiently uncomplicated, defining just 243 unique states, to permit these kinds of analysis to be undertaken [5].

The EQ-5D-3L is a measure of self-reported health-related quality of life, accompanied by weights reflecting the relative importance to people of different health states. It was designed to be a brief questionnaire that is generic, meaning that it measures health in a way that can be compared across different patient demographics, health statuses, disease areas and treatments, and minimises the burden of data collection. The questionnaire collects data from respondents on five aspects of their HRQOL, called dimensions: Mobility; Self-Care; Usual Activities; Pain & Discomfort; and Anxiety & Depression. Respondents select one of three levels to describe problems that they have in each dimension, described as no, some or extreme problems in the Pain & Discomfort and Anxiety & Depression dimensions and no, some and inability to undertake in the Mobility, Usual Activities and Self-care dimensions. The questionnaire also contains a Visual Analogue Scale, called the EQ VAS, on which respondents record their overall assessment of their health on a scale from 0 (worst possible health state) to 100 (best possible health state). The layout of the questionnaire can be seen in the EQ-5D-3L User Guide, obtainable from the EuroQol website (https://euroqol.org). There is a newer version of this, the EQ-5D-5L, which has five levels within each of the dimensions [6], but this version was not used in the studies reported here.

The combination of levels in each dimension describes an EQ-5D self-reported health state, often called an ‘EQ-5D profile’. Each profile can be described as five sentences that identify the level within each dimension, for example: No problems in walking about; No problems with self-care; Some problems with performing usual activities; Extreme pain or discomfort; Moderate anxiety or depression. A simpler way to summarise a profile is to assign each level a number and describe the profile as a 5-number string, representing the level of each dimension in the order in which they appear in the questionnaire. The numbers used are: Level 1 = no problems; Level 2 = some problems; and Level 3 = extreme problems or unable to. So, for example, no problems in any dimension is 11111, some problems in every dimension is 22222, and extreme problems in every dimension is 33333. The profile described above would be 11232. Note that although the numbers assigned to levels within dimensions are ordinal - 3 is worse than 2 and 2 is worse than 1 - the profile labels are categories, not numbers, and do not have full ordinal properties [7].

Neither the EQ-5D-3L nor the EQ-5D-5L have a single algorithm for calculating an index number, but essentially algorithms give a value to each profile by assigning a weight to each response within each dimension and using a mathematical formula to combine them. The weights themselves are usually derived from studies of population preferences for different EQ-5D-3L or EQ-5D-5L profiles. From these, a list of values for each profile can be generated, called ‘value sets’; there are many such sets, usually based on the preferences of a country’s general population [8, 9].

This paper draws on the methods described by Devlin, Parkin and Janssen [10] to explore the value of analysing data in the form of patient responses to the EQ-5D-3L questionnaire, complementary to their role in generating values for use in cost-effectiveness analyses, including showing the limitations of this approach. These cover analyses based on:

1. Frequency distributions of levels and dimensions at different points in time.
2. Frequency distributions of changes in levels within dimensions over time.
3. Comparing between different groups frequency distributions of levels and changes at different points in time and of changes over time.
4. Changes in profiles using the Paretian Classification of Health Change and the Health Profile Grid.
5. Frequency distributions of common profiles, compared over time and between groups.
6. Combining descriptive and EQ VAS data.

## Data and methods

The study was a post-hoc analysis of EQ-5D-3L data from two prospective, non-randomised, single arm clinical studies of two knee replacement systems, the ATTUNE® Knee and SIGMA® Knee (both DePuy Synthes, Warsaw, IN, USA) [11]. Patients were operated on by the same surgeons and implanted with the devices between 2011 and 2015 across 24 sites and four countries (USA, UK, Australia and New Zealand). Both Attune and Sigma patients were studied in 18 sites, with Attune patients only in five additional sites and Sigma only in one. As well as the EQ-5D-3L, three other PROMs were collected: the Oxford Knee Score (OKS), the Knee Injury and Osteoarthritis Outcome Score (KOOS) [12], and the Patient’s Knee Implant Performance (PKIP) [13].

The data are longitudinal, with observations at four points: pre-surgery; up to 10 months post-surgery, described in the text as ‘10 months or less’ and labelled ‘ < 11 months’ in tables; 11–22 months; and 23–34 months, labelled ‘ > 22 months’ in tables. However, not all of the 1879 patients who completed a questionnaire did so at every point. Looking first at those who completed a profile, there were 1875 pre-surgery, 1793 (96%) at 10 months or less, 1631 (87%) at 11–22 months and 1563 at 23–34 months (83%). This gives a reasonable overall response rate of 89%. Most of the missing profiles were because the patient did not complete a questionnaire at all. There were however small numbers of patients who missed one dimension and were therefore excluded; 2 pre-surgery, 1 at 10 months or less, 4 at 11–22 months and 5 at 23–34 months. It was therefore necessary to define a consistent set of data for analysis. First, each of the cross-sectional observations was checked for evidence of selection bias, for example whether patients with worse or better health were more or less likely to complete follow-up questionnaires. The proportion of patients who had had at least some problems pre-surgery was slightly larger at 10 months or less than at 11–22 months and at 23–34 months. The differences were small, so it is unlikely that this will have a large impact on the results, but this should be considered when assessing the results of the analyses.

Secondly, the distributions of all EQ-5D variables for the ‘11–22 months’ and ‘23–34 months’ data were very similar, but different to the ‘10 months or less’ data. It is therefore not necessary to include results for the ‘23–34 months’ questionnaire here, but this in itself is a substantive finding: there were shorter term improvements following surgery at 10 months or less and continuing improvements over the following year, but no further improvements, or deterioration, for the subsequent year.

In analysing changes over time, our profile analyses therefore cover those who completed questionnaires pre-surgery and at 10 months or less (1789), those who completed questionnaires pre-surgery and at 11–22 months (1627) and those who completed questionnaires at all three points (1587). These populations were used to include the largest sample size available at each timepoint.

There were also small numbers of patients who completed a profile but who did not complete the EQ VAS and were therefore excluded from analyses that used those data: 3 pre-surgery, 1 at 10 months or less, 1 at 11–22 months and 6 at 23–34 months. The analysis reported below uses data from patients who completed questionnaires pre-surgery and at 11–22 months; of the 1827 who completed a profile in both of those years, 3 did not complete the EQ VAS, giving a sample size of 1624 for those analyses.

The data were analysed using R version 3.5.3 (“Great Truth”) and Microsoft Excel™ to generate frequency and contingency tables and to undertake regression analyses of the relationship between EQ VAS values and EQ-5D profiles. This should be viewed as exploratory data analysis, complementary to studies that aim to explore hypotheses about health and how it is affected by health care. They therefore do not involve inference techniques such as statistical tests of differences between patients according to different environments.

## Results

Most of the tables reported in this paper are for all patients, irrespective of the knee replacement system that was implanted, except where the point of the analysis is to show how comparisons between different procedures may be presented. Apart from these exceptions, comparisons between Attune and Sigma patients are noted, and a data supplement contains the separate tables for them.

### Dimensions and levels

#### Overall quality of life

**Fig. 1:**
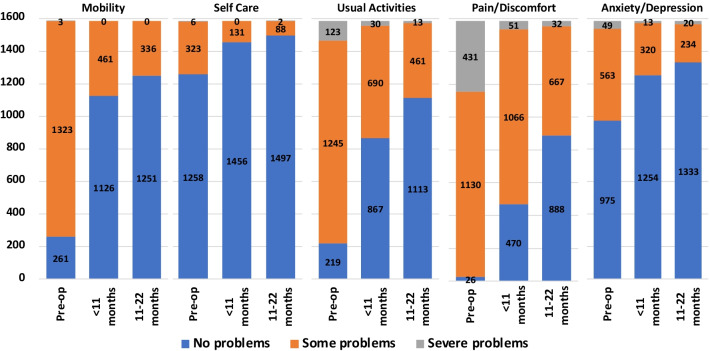
Number of TKA patients by level within EQ-5D-3L dimensions at different observation points (*N* = 1587)

Figure 1 shows the number of respondents (including both Attune and Sigma patients, *N* = 1587) in each level of each EQ-5D-3L dimension at different observation points, for patients who completed all 3 questionnaires. Numbers and percentages are shown in Additional file 1: Table S1. The distributions of pre-surgery levels give a picture of the amount of unmet need for patients undergoing surgery. Pain & Discomfort was the most frequently observed problem: 1.6% of patients reported no problems (Level 1), 27.2% severe problems (Level 3) and the remainder some problems (Level 2). Usual Activities was the second most frequent: 13.8% no problems, 7.8% severe problems and the remainder some problems. The mobility dimension also had less than half of patients reporting no problems (16.4%), 83.3% had some problems, but only 0.2% reported severe problems. This is likely to be due to a known limitation of the EQ-5D-3L Mobility Level 3, which labels this “Confined to bed” [14]. This ceiling effect issue has been corrected in the EQ-5D-5L [6], where the most severe problem with mobility is “Unable to walk about” and there is an option for “Severe problems in walking about”. More than half of patients reported no problems for Anxiety & Depression (61.4%) and Self-care (79.3%), and relatively small numbers reported extreme problems (3.1% and 0.4%); in each case the remainder had some problems.

The distributions of post-surgery levels show marked improvements in every dimension. However, the point at which this was observed differed between dimensions. For Mobility, Self-Care and Anxiety & Depression, most of the improvement was seen in the first 10 months (pre-surgery to 10 months or less), with small further improvements in the following year (10 months or less to 11–22 months). For Usual Activities and Pain & Discomfort, there was a large improvement in the first 10 months and a further large improvement in the following year. In both cases, the second-year improvements were an increase in the number of patients reporting no problems, rather than a reduction in the number of patients reporting severe problems.

For Pain & Discomfort, the most frequently observed problem pre-surgery, 29.6% reported no problems during the first 10 months after surgery, rising to 56.0% in 11–22 months. Only 2% had severe problems in 11–22 months. Most of the improvement in Pain & Discomfort occurred in the first 10 months. There was a similar pattern for the second most frequently reported problem, Usual Activities, with no problems rising to 54.6% during the first 10 months and to 70.1% in 11–22 months, and severe problems falling to 1.9% and subsequently to 0.8%. For Mobility, 71% had no problems during the first 10 months, rising to 78.8% in 11–22 months.

Although there were large improvements in the dimensions most associated with the underlying condition, post-surgery there remained (even in 11–22 months) a significant number of patients experiencing problems with Mobility, Usual Activities and, in particular, Pain & Discomfort. For the other two dimensions, there were also large improvements, although from a lower level of pre-surgery problems. 5.6% of patients reported problems with Self Care post-surgery, and 16.0% with Anxiety & Depression; these may represent an underlying level of problems consistent with the general population.

#### Comparison of attune and sigma patients

Additional file 1: Tables S2 and S3 show the overall pattern of levels within dimensions and changes over time for Attune and Sigma patients, which were very similar to each other, with the exception of Mobility. Both pre- and post-surgery, Sigma patients had fewer mobility problems than Attune patients, reflecting a slightly different pre-surgery case mix. Pre-surgery, more Attune (87.5%) than Sigma (77.4%) patients reported Level 2 problems and three Attune patients reported Level 3 problems, compared with none for Sigma. At 11–22 months, the percentages with Level 2 problems were 23.8% and 17.4% respectively; there were no patients with Level 3 problems in either the Attune or Sigma group.

Table 1 compares between Attune (*N* = 937) and Sigma (*N* = 650) patients the increases in the percentage of patients reporting no problems within each dimension in the first- and second- years post-surgery compared with pre-surgery. It shows that in every dimension, the improvement was greater in the second-year post-surgery for Attune than Sigma, including Mobility. Except for Usual Activities, the same is the case in the first-year post-surgery.

**Table 1:** Comparison between knee replacement systems of increase in percentage of patients reporting no problems within EQ-5D-3L dimensions at different observation points

	< 11 months		11–22 months	
	Attune	Sigma	Attune	Sigma
Mobility	55.7	52.8	64.0	60.0
Self care	13.6	10.9	16.8	12.6
Usual activities	40.3	41.5	57.3	54.9
Pain & discomfort	28.3	27.5	56.6	51.1
Anxiety & depression	18.4	16.5	24.9	19.2

#### Changes in levels within dimensions

Table 2 shows the pattern of changes between the different time points (*N* = 1587). There are three possible start and end levels, so there are 9 possible pathways of change within each dimension. These can be divided into *improvement* pathways (2–1, 3–1 and 3–2), *worsening* pathways (1–2,1–3 and 2–3) and *stable* pathways (1–1, 2–2 and 3–3). In the table, improvement pathways are highlighted in bold and worsening pathways in italics. The first pair of data columns show the number and percentage in each change pathway, measured from the pre-surgery baseline to the first post-surgery assessment (< 1 year). The second pair show the changes from the pre-surgery baseline to the second post-surgery assessment (> 1 year). The third pair show the changes from the first post-surgery assessment to the second. The third pair are therefore not directly comparable with the other pairs, because they start from a different baseline.

**Table 2:** Changes over time in Levels within dimensions

	Change	Pre- to < 11 months		Pre- to 11–22 months		< 11 months to 11–22 months	
		*N*	%	*N*	%	*N*	%
Mobility	1–1	235	90.0	251	96.2	979	86.9
	*1–2*	*26*	*10.0*	*10*	*3.8*	*147*	*13.1*
	*1–3*	*0*	*0.0*	*0*	*0.0*	*0*	*0.0*
	**2–1**	**889**	**67.2**	**998**	**75.4**	**272**	**59.0**
	2–2	434	32.8	325	24.6	189	41.0
	*2–3*	*0*	*0.0*	*0*	*0.0*	*0*	*0.0*
	**3–1**	**2**	**66.7**	**2**	**66.7**	**0**	
	**3–2**	**1**	**33.3**	**1**	**33.3**	**0**	
	3–3	0		0		0	
Self-care	1–1	1205	95.8	1222	97.1	1409	96.8
	*1–2*	*53*	*4.2*	*35*	*2.8*	*46*	*3.2*
	*1–3*	*0*		*1*	*0.1*	*1*	*0.1*
	***2–1***	**246**	**76.2**	**269**	**83.3**	**88**	**67.2**
	2–2	77	23.8	53	16.4	42	32.1
	*2–3*	*0*	*0.0*	*1*	*0.3*	*1*	*0.8*
	**3–1**	**5**	**83.3**	**6**	**100.0**	**0**	
	**3–2**	**1**	**16.7**	**0**	**0.0**	**0**	
	3–3	0	0.0	0	0.0	0	
Usual activities	1–1	171	78.1	196	89.5	741	85.5
	*1–2*	*48*	*21.9*	*23*	*10.5*	*124*	*14.3*
	*1–3*	*0*	*0.0*	*0*	*0.0*	*2*	*0.2*
	**2–1**	**647**	**52.0**	**853**	**68.5**	**358**	**51.9**
	2–2	575	46.2	382	30.7	322	46.7
	*2–3*	*23*	*1.8*	*10*	*0.8*	*10*	*1.4*
	**3–1**	**49**	**39.8**	**64**	**52.0**	**14**	**46.7**
	**3–2**	**67**	**54.5**	**56**	**45.5**	**15**	**50.0**
	3–3	7	5.7	3	2.4	1	3.3
Pain & discomfort	1–1	14	53.8	19	73.1	374	79.6
	*1–2*	*12*	*46.2*	*7*	*26.9*	*95*	*20.2*
	*1–3*	*0*	*0.0*	*0*	*0.0*	*1*	*0.2*
	**2–1**	**359**	**31.8**	**674**	**59.6**	**505**	**47.4**
	2–2	749	66.3	445	39.4	539	50.6
	*2–3*	*22*	*1.9*	*11*	*1.0*	*22*	*2.1*
	**3–1**	**97**	**22.5**	**195**	**45.2**	**9**	**17.6**
	**3–2**	**305**	**70.8**	**215**	**49.9**	**33**	**64.7**
	3–3	29	6.7	21	4.9	9	17.6
Anxiety & depression	1–1	892	91.5	915	93.8	1160	92.5
	*1–2*	*82*	*8.4*	*59*	*6.1*	*90*	*7.2*
	*1–3*	*1*	*0.1*	*1*	*0.1*	*4*	*0.3*
	**2–1**	**342**	**60.7**	**393**	**69.8**	**171**	**53.4**
	2–2	212	37.7	157	27.9	137	42.8
	*2–3*	*9*	*1.6*	*13*	*2.3*	*12*	*3.8*
	**3–1**	**20**	**40.8**	**25**	**51.0**	**2**	**15.4**
	**3–2**	**26**	**53.1**	**18**	**36.7**	**7**	**53.8**
	3–3	3	6.1	6	12.2	4	30.8

Bold = improvements, Italics = worsening, no bold or italic = no change

This table adds more detail to the finding that most improvements occurred in the first-year post-surgery, although in some dimensions there are also substantial improvements in 11–22 months. In every dimension there were few patients in worsening pathways by 11–22 months: the largest percentage was in Anxiety & Depression, where 73 out of 1587 patients were in these pathways (4.6%); the others were in decreasing order Self Care (2.33%), Usual Activities (2.27%), Pain & Discomfort (1.13%) and Mobility (0.6%). Moreover, few patients who had no problems pre-surgery developed problems after surgery. In every dimension the majority of patients who had at least some problems (that is, Level 2 or Level 3) 11–22 months post-surgery had also had at least some problems in that dimension pre-surgery: in decreasing order Pain & Discomfort (98.9%), Mobility (97.0%), Usual Activities (95.1%), Anxiety & Depression (76.7%) and Self Care (65.5%).

Additional file 1: Tables S4, S5 and S6 compare improvement and worsening pathways between Attune and Sigma. In the first 10 months post-surgery there was a mixed pattern of the relative numbers of patients in improvement pathways and worsening pathways; however it should be remembered that there were few patients in worsening pathways in either group, so the differences between them are small. For Self-Care, there was a slightly larger percentage (1.9%) of Attune patients in improvement pathways and a slightly smaller percentage (− 0.86%) in worsening pathways; for Mobility and Anxiety & Depression there were also more Attune patients in improvement pathways (3.2% and 2.8%) but also more in worsening pathways (0.17% and 0.18%); for Pain & Discomfort there were fewer Attune patients in both improvement (− 1.4%) and worsening (− 0.54%) pathways; and for Usual Activities there were fewer Attune patients in improvement pathways (− 1.9%) and more in worsening pathways (1.8%). However, after 11–22 months, there were in every dimension a slightly greater percentage of Attune patients in improvement pathways and a slightly smaller percentage in worsening pathways; for example, 3.4% more improving and 0.76% fewer worsening in Mobility.

### Analysing changes in profiles

#### Change categories

Looking at how levels within dimensions change in the distribution of EQ-5D-3L health states is valuable, but it does not give a picture of how individual patients’ health states as a whole change. The Paretian Classification of Health Change (PCHC) [5] summarises these by classifying a patient’s health state into four categories:

- Improve - an improvement in at least one dimension and no deterioration in any other dimension. For example, pre-surgery 21321; post-surgery 21221.
- Worsen - a deterioration in at least one dimension and no improvement in any other dimension. For example, pre-surgery 21321; post-surgery 31321.
- No change - no change in any dimension. For example, pre-surgery 21321; post-surgery 21321.
- Mixed - an improvement in at least one dimension and a deterioration in at least one dimension. For example, pre-surgery 21321; post-surgery 22221.

Table 3 shows the distribution of change from pre-surgery to the first 10 months (*N* = 1789) and 11–22 months (*N* = 1627) post-surgery. 76% of patients improved over the first 10 months, rising to 85% in 11–22 months. This improvement of the patient group as a whole resulted from a reduction in all of the other three categories: the number who worsened fell from 7.5% in the first 10 months to 3.5% in 11–22 months; those whose quality of life did not change fell from 9.2 to 5.9%; and those who had a mixed change fell from 7.8 to 5.5%. Additional file 1: Table S7 compares the PCHC between Attune and Sigma patients. At both first- and second- years post-surgery a greater percentage of Attune patients improved and fewer had a mixed change, and by 11–22 months fewer also worsened and more had no change.

**Table 3:** Paretian classification of health change from pre- to post-surgery, for all patients

	Pre-surgery to < 11 months		Pre-surgery to 11–22 months	
	*N*	%	*N*	%
Improve	1350	75.5	1385	85.1
Worsen	134	7.5	57	3.5
No change	165	9.2	96	5.9
Mixed	140	7.8	89	5.5
Total	1789		1627	

The characteristics of those who either worsened or experienced no change according to the PCHC were further investigated by examining their profiles and for those who worsened the specific dimensions in which they worsened.

For patients who experienced no change, there was no evidence that this was related to any particular profile. The most common profiles for such patients, both Attune and Sigma, were those that were most common in patients as a whole pre-surgery (see analysis of profiles below).

For patients whose health state worsened (*N* = 134 to < 11 months, *N* = 57 to 11–22 months), Table 4 shows the number of dimensions in which they became worse. In both the first- and second- years post-surgery, of the patients who worsened, the large majority worsened in one dimension only (75.4% and 70.2% respectively). Although the number of patients who became worse in more than one dimension fell to almost half (33–17) in 11–22 months, they formed a slightly larger percentage of the total (24.6–29.8%). Additional file 1: Table S8 compares Attune and Sigma patients. In the first 10 months there were slightly more Attune patients with larger numbers of worse dimensions (25.2–23.5%), but in 11–22 months there were far fewer (20–40.7%).

**Table 4:** Number of worse dimensions for patients whose quality of life worsened, for all patients

Number of worse dimensions	Pre-surgery to < 11 months		Pre-surgery to 11–22 months	
	*N*	%	*N*	%
1	101	75.4	40	70.2
2	22	16.4	10	17.5
3	9	6.7	5	8.8
4	2	1.5	2	3.5

Table 5 shows for these patients who became worse overall the dimensions in which they worsened. In both the first- and second- years post-surgery, the smallest numbers were in those dimensions in which there were most problems pre-surgery; Mobility and Pain & Discomfort. Although there were differences between the other dimensions in the number of patients in the first 10 months, in 11–22 months they were the same. Additional file 1: Table S9 compares Attune and Sigma patients. Attune patients followed the same pattern as patients overall, but there were some differences for Sigma patients. The relatively small numbers in each patient group make detailed interpretation difficult, but for Sigma patients Pain & Discomfort worsening in the first 10 months post-surgery was as prevalent as in other dimensions apart from Mobility. The reduction in numbers worsening in both Mobility and Pain & Discomfort was much less apparent for Sigma patients, such that the percentage who worsened in Mobility, which was much smaller in 11–22 months compared with the first for Attune patients (12–6.7%), rose (11.8–18.5%).

**Table 5:** Dimensions that worsened for patient who worsened overall

EQ-5D dimension	Pre-surgery to < 11 months		Pre-surgery to 11–22 months	
	*N*	%	*N*	%
Mobility	16	11.9	7	12.3
Self-care	33	24.6	22	38.6
Usual activities	47	35.1	22	38.6
Pain & discomfort	26	19.4	10	17.5
Anxiety & depression	58	43.3	22	38.6

Percentages are of the number of patients who worsened (*N* = 134 to < 11 months, *N* = 57 to 11–22 months)

#### Health profile grid

**Fig. 2:**
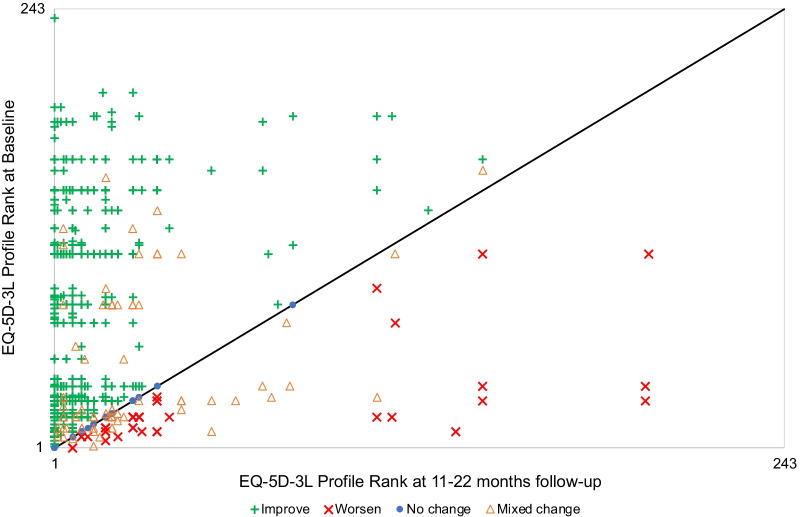
Comparison of EQ-5D-3L profiles at baseline and at 11–22 months

The Health Profile Grid [5] compares each patient’s profile at baseline with their profile at follow-up, in this case at 11–22 months (*N* = 1627). The profiles are arranged in rank order from best to worst defined by a value, in this case the US Time trade off (TTO)-based Value Set. Figure 2 shows these pairings. The black diagonal line shows the points at which baseline- and follow-up profiles are the same, in other words the patient experienced no improvement or deterioration in any dimension of the EQ-5D. Points above the line represent improvements, and points below a deterioration. (Note that gaps between apparent clusters of patients and points apparently in straight lines are artefacts of the method and have no real interpretations in terms of differences between patients.) The points are also identified by their change category according to the PCHC, which in effect identifies those who had a Mixed Change.

As expected, a large majority of patients (1439/1627 = 88.4%) are above the line. There are more patients who experienced no change (96/1627 = 5.9%) than a deterioration (92/1627 = 5.7%). Additional file 1: Figure S1 compares Attune and Sigma patients. A larger percentage of Attune patients improved (89.3% Attune, 87.3% Sigma) and had no change (6.2% Attune, 5.5% Sigma), and therefore a smaller percentage deteriorated (4.5% Attune, 7.3% Sigma).

### Common profiles

Table 6 shows the most common EQ-5D-3L profiles observed pre-surgery and at follow up for patients who completed questionnaires at both < 11 months and > 1 year follow-up as well as pre-surgery (*N* = 1587). As is usual with EQ-5D-3L data, a small proportion of all of the possible profiles describe a large proportion of the observations. The table shows the most frequent profiles that describe 90% of the observations.

**Table 6:** The most common EQ-5D-3L profiles observed pre-surgery and at follow up, for all patients

Pre-surgery				< 11 months				11–22 months			
Profile	*N*	%	Cu%	Profile	*N*	%	Cu%	Profile	*N*	%	Cu%
21221	409	25.8	25.8	11111	350	22.1	22.1	11111	716	45.1	45.1
21222	188	11.8	37.6	11121	345	21.7	43.8	11121	234	14.7	59.9
21231	118	7.4	45.1	11221	199	12.5	56.3	21221	113	7.1	67.0
11221	100	6.3	51.4	21221	147	9.3	65.6	11221	88	5.5	72.5
21232	92	5.8	57.2	21222	82	5.2	70.8	11211	60	3.8	76.3
11121	81	5.1	62.3	11122	53	3.3	74.1	11112	40	2.5	78.8
22221	77	4.9	67.1	11222	53	3.3	77.4	21222	40	2.5	81.3
22222	74	4.7	71.8	11211	48	3.0	80.5	11122	36	2.3	83.6
21121	69	4.3	76.1	21121	46	2.9	83.4	11222	36	2.3	85.9
22232	58	3.7	79.8	22222	40	2.5	85.9	22222	27	1.7	87.6
22231	36	2.3	82.0	22221	37	2.3	88.2	21121	26	1.6	89.2
22332	31	2.0	84.0	11112	29	1.8	90.0	21211	22	1.4	90.6
11222	27	1.7	85.7								
21122	22	1.4	87.1								
21321	17	1.1	88.2								
21332	17	1.1	89.2								
21223	14	0.9	90.1								

Pre-surgery, 4 profiles accounted for 50% of all observations and 17 for 90% of them, all of which include some or severe Pain & Discomfort. The most common, accounting for over a quarter of all patients, is 21221: some problems with Mobility, Usual Activities and Pain & Discomfort and no problems with Self Care and Anxiety & Depression. The other profiles differ by having a more severe level in one or more dimensions, though four of them have a less severe level in one or both of Mobility and Usual Activities.

Post-surgery, 12 profiles account for 90% of observations at both less than and greater than 11 months follow up; these are the same profiles at each time point with one exception amongst less common observations – a more severe condition, 22221, replaced by less severe, 21211 – and other more severe conditions are relatively less frequent. The profiles are even more concentrated than pre-surgery. The most common profile 11,111 – no problems in any dimension – covers 22.1% and 45.1% of observations at less than and greater than 11 months; at least 50% of observations are covered by 3 and 2 profiles respectively.

Additional file 1: Tables S10 and S11 show the most frequently observed pre-surgery profiles for Attune and Sigma patients. These were mostly the same, except for some of the less common profiles, 21122 and 21223 (in Attune but not Sigma) and 11222 (Sigma but not Attune). However, more severe profiles were relatively more frequent in Attune than Sigma; the top 50% for Sigma included the less severe profile 11221 instead of 22211 and the less severe profiles 11221 and 11121 were relatively more frequent in Sigma (16% of all profiles) than Attune (8%). This finding is consistent with that from "Results" section, that there were slightly greater mobility problems amongst Attune patients pre surgery.

Post-surgery, the most common profiles were again very similar. Both had 11111 and 11121 covering 50% of patients, although a slightly greater percentage of Attune patients were in the ‘full health’ profile 11111. In contrast to the pre-surgery distribution, where there were relatively more severe profiles amongst Attune patients, post-surgery more severe profiles were slightly more frequently observed amongst Sigma patients.

### Combining EQ-5D descriptive system and EQ VAS data

As well as data in the form of the EQ-5D descriptive system, the EQ-5D questionnaire provides data in the form of the EQ VAS. Using these data together can provide further insights into respondents’ HRQOL. Two examples are given here: the relationship between profiles and the EQ VAS, and the relationship between change categories and the EQ VAS.

#### The relationship between profiles and the EQ VAS

Information on the distribution of levels and dimensions over a patient population demonstrates their relative importance in terms of prevalence within that population. An equally valuable question is how important they are to patients. Examining the relationship between the profiles and the EQ VAS gives an indicator of this.

Regression analysis using Ordinary Least Squares was used to estimate the impact of each level and dimension of the EQ-5D on the EQ VAS. Table 7 summarises the results, pre- and > 11 months post-surgery (*N* = 1624). There were so few observations for Level 3 (extreme problems) on Mobility and Self Care that it was not possible to identify an impact separate to that of Level 2 (some problems); for Pain & Discomfort pre-surgery it was only possible to identify Level 3 effects. All other levels and dimensions had the expected pattern, that is their coefficients were negative and their Level 3 coefficients were greater than Level 2 coefficients.

**Table 7:** Regression coefficients for EQ VAS with EQ-5D-3L dimensions and levels for all patients

	Pre-surgery		Post-surgery	
	Estimate	Std. error	Estimate	Std. error
(Intercept)	91.47	3.08	87.92	0.42
Mobility 2	− 4.30	1.13	− 5.86	0.93
Mobility 3	*− 8.04*	9.98	*NA*	*NA*
Selfcare 2	− 4.38	1.01	− 5.53	1.42
Selfcare 3	*2.49*	6.53	*3.87*	8.78
Usual activity 2	− 4.14	1.21	− 5.88	0.85
Usual activity 3	− 9.49	1.88	− 20.37	3.27
Pain & discomfort 2	*− 5.85*	3.11	− 2.61	0.71
Pain & discomfort 3	− 8.96	3.22	− 10.97	2.39
Anxiety & depression 2	− 7.11	0.84	− 8.03	0.91
Anxiety & depression 3	− 14.81	2.26	− 16.10	2.74
Adjusted R^2^	0.171	0.335		

Italic = Not significantly different from zero at 0.05 level, or cannot be computed (NA)

Although the relative sizes of coefficients were very similar pre- and post-surgery, as described below, the size of all of the coefficients that were significantly different from zero were higher post-surgery. The goodness of fit, as measured by R^2^, was also higher.

The dimension that had the most impact overall on measured quality of life was Anxiety & Depression. It had the largest Level 2 coefficient both pre- and post-surgery, and Level 3 was the largest pre-surgery and the second largest post-surgery. Mobility, Self-Care and Usual Activities had similar coefficients at Level 2 both pre- and post-surgery, but post-surgery the Usual Activities Level 3 coefficient was much higher than pre-surgery and was the largest of the post-surgery coefficients. The Pain & Discomfort coefficients were consistently the smallest of the significant coefficients, at both Level 2 and Level 3 and both pre- and post-surgery.

Additional file 1: Tables S12, S13 and S14 compare Attune and Sigma patients. A dummy variable for knee replacement system included in the all-patients analysis had a coefficient in each year’s equation that was not significantly different from zero and did not change goodness of fit as measured by adjusted R^2^. However, analysing Attune and Sigma patients data separately, the relative sizes of the coefficients, both for dimension levels and for pre- and post-surgery, were in some cases different. Two examples are that Mobility at Level 2 had a slightly smaller coefficient for Attune patients post-surgery than pre-surgery, but for Sigma patients it was very much larger; and pre-surgery Self-Care had the smallest coefficient for Attune patients, for Sigma patients it was Mobility.

#### The relationship between change categories and the EQ VAS

The Paretian change categories provide a simple indicator of how successful are interventions such as a TKA. It is valuable to explore factors that might affect that success. A proper assessment of this would only be possible by examining a wide range of factors affecting patients before, during and after surgery, but using the pre-surgery EQ VAS gives an insight into one factor, the severity of the patient’s condition as measured by their reported HRQOL.

**Fig. 3:**
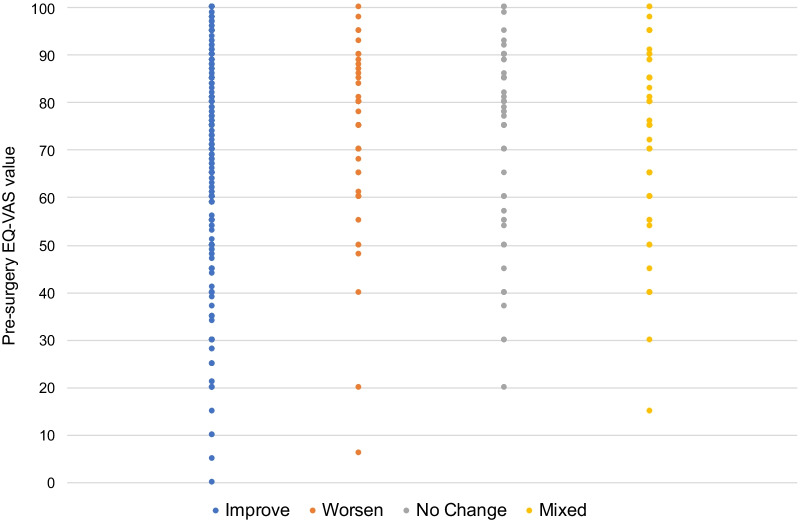
Pre-surgery EQ VAS score by change category, for all patients

The distributions of pre-surgery EQ VAS values for the four change categories are very similar in terms of summary statistics (pre- and > 11 months post-surgery, *N* = 1624). Means, with standard deviations in parentheses, are Improved 73.3 (17.3), Worsened 72.7 (17.4), No change 74.0 (17.5) and Mixed Change 72.1 (16.3). This can be further explored by visual examination of the distributions, as in Fig. 3. Each dot represents one patient. It shows that those who improved recorded pre-surgery EQ VAS values over the full range of possible values. For the other categories, there was a similar spread over the range of possible values, although with a lower minimum value, because for the most severe conditions, there is less scope for worsening.

## Conclusions

This paper has demonstrated the value of analysing the patient’s responses to PROMs questionnaires without using summary scores derived from those responses. In this case, the results add further detail to existing knowledge about the significant health problems that TKA patients experience before surgery, the improvements that result from surgery, the residual problems that a minority have, and the time periods over which improvements are experienced.

In this sample of patients, their pre-surgery unmet needs were identified, by both the analysis of levels and dimensions and profiles as a whole, as problems mainly with suffering pain or discomfort and restrictions on mobility and being unable to undertake their usual activities. As a result, a small number of descriptions of their overall health, in the form of EQ-5D profiles, are observed including some problems with each of these problems but no problems with caring for themselves, anxiety or depression.

Another finding for this sample that was consistent between the different ways in which the data were analysed was that the largest improvements after surgery were in those dimensions identified as the most prevalent pre-surgery; but even so some patients continued to experience problems in these dimensions, especially in pain and discomfort. Few patients worsened overall after surgery, most of those that did became worse in one dimension only, and they worsened mainly in caring for themselves, undertaking their usual activities and experiencing anxiety or depression. One explanation for this is that mobility, pain and discomfort are directly dealt with by the surgical procedure, and because of good TKA success rates it is relatively rare to find patients who become worse in these dimensions. The other three dimensions are more dependent on external personal and social factors and only indirectly addressed by surgical intervention.

Collecting data at two follow-up points meant that insights could be gained not only regarding levels of improvement in health overall, but also about the speed at which improvements were gained. The different methods that we used to analyse this issue were again consistent in their conclusions. Improvements in the ability to undertake usual activities and reductions in pain and discomfort continued over 11–22 months, while for the other dimensions of health the improvements were mainly in the first 10 months.

The analysis of changes over time in levels within dimensions given in Table 2 above may represent the outer limit of what is possible with these kinds of analysis. Although interpretation of the table is straightforward, its complexity means that it is difficult to detect and summarise patterns within the multiple comparisons involved. The addition of further dimensions, levels or time periods would multiply this problem to a point where the aim of undertaking relatively simple analyses is no longer met.

## Discussion

The added value that these analyses generate arises from their ability to examine closely different dimensions of ill health. It is often assumed that the ability to obtain such insights is a feature of condition-specific PROM instruments because they ask many questions about different aspects of health, each of which have multiple answer options. Our study suggests that this advantage over simpler generic instruments may be overstated, although this does not mean that condition-specific measures are not needed; it remains best practice for the collection of PROMs where possible to collect data from both condition-specific and generic instruments, as these provide complementary information. The EQ-5D is very easy to use from the point of view of both those who want to collect PROMs data and those from whom the data are collected but can yield much valuable information if the full content of the data is analysed. In the work reported here, the different ways of analysing the data gave consistent messages, but each gave different insights.

Analysis of EQ VAS values offers a useful view of patients’ overall health-related quality of life, but these data are even more valuable when analysed with the profile data. For example, our analysis suggests it is not possible to predict from the severity of a patient’s pre-surgery condition in terms of their overall quality of life, as measured by the EQ VAS, to predict the direction that quality of life, as assessed by the EQ-5D profile, will change. This suggests that pre-surgery assessment of HRQOL, at least that measured by the EQ VAS, would not add to those assessments of the likely success of a TKA already used by clinicians.

The other advantage claimed for these analyses is that they rely only on actual responses by patients and are therefore strictly patient reported. Most instruments that generate index numbers use externally defined scoring systems that reflect the relative importance of different responses. (An exception is the SEIQOL family of instruments [15], which use values obtained from patients to weight their own responses.) This is an argument in favour of the methods discussed here but does not imply that weighting systems are in some way misleading. It is essential to use an external scoring system if the aim is to analyse data in conformity with the aims for which the system was created.

However, the EQ-5D provides an alternative way of looking at the relative importance to patients of its different levels and dimensions by combining it with EQ VAS data using regression analysis. This is a different measure of how important dimensions and levels to that measured by the frequency with which they are observed in the patient group as a whole. It shows the impact of different aspects of patients’ quality of life on their overall self-assessment of it, not just on how many patients have those problems. Because patients report both the EQ VAS and their profile, this measure of importance is entirely patient reported.

The results from this study illustrate the importance of considering both of these measures of importance. Although problems with pain or discomfort are very frequently reported both before and after surgery, this may have a relatively small impact on overall quality of life; by contrast, anxiety and depression problems are less often reported, but have a very large impact on overall quality of life. The larger impact of problems that patients have with undertaking their usual activities after surgery compared with before may reflect disappointment with unrealised expectations for the results of the surgery. Patient education conducted pre-surgery could help manage post-surgical expectations.

Two factors limit the extent to which we can draw definite conclusions from analysis of EQ-5D-3L data for knee patients. First, there is the known limitation of the EQ-5D-3L Mobility Level 3 response description “Confined to bed”, an extreme case unlikely to be experienced by most of those who have greater mobility problems than the description “Some problems” [14]. (As noted, this issue has been corrected in the EQ-5D-5L.) Secondly, because the EQ-5D does not identify the site or cause of any problems, it is possible that some of the residual mobility and pain or discomfort issues may not relate to the knee itself but to elsewhere in the body.

A limitation of the study is that no inferential statistical analyses were conducted due to the level of complexity required to undertake comparisons across dimensions, levels and timepoints. No adjustments were made for differences in baseline characteristics between the two implant groups for the same reason. However, the results show clear patterns in the data that could be used as the basis of hypothesis testing and estimation of effects.

A final limitation of our study is that it only investigates one PROM instrument, the EQ-5D-3L, and not others potentially capable of being analysed in this way such as the SF-6D [16]. As noted above, it is possible that many instruments may be too complex to be capable of many of the analyses reported here.

The true potential value of the analytical methods used arises from implications of the results for improvements in the patient pathway. The results reported in this study could be used to ensure the tailored, holistic care of the patient throughout their TKA experience. For example, it is important to assess the patient’s pre-surgery assessment across the 5 dimensions of the EQ-5D-3L to ensure that appropriate measures are put in place to manage areas that have been shown to have a very large impact on overall quality of life, like anxiety and depression. The benefits shown of Attune over Sigma also highlight the role that implant selection could play in patient outcomes such as quality of life improvements.

## Supplementary Information

**Additional file 1**: **Table S1**. Number of patients by level within EQ-5D-3L dimensions at different observation points (all patients). **Table S2**. Number of Attune patients by level within EQ-5D-3L dimensions at different observation points. **Table S3**. number of Sigma patients by level within EQ-5D-3L dimensions at different observation points. **Table S4**. Changes over time in Levels within dimensions for Attune patients. **Table S5**. Changes over time in Levels within dimensions for Sigma patients. **Table S6**. Changes over time in levels within dimensions, comparing Attune and Sigma. **Table S7**. Classification of Change from pre- to post-surgery, comparing Attune and Sigma. **Table S8**. Number of worse dimensions for patients whose quality of life worsened, comparing Attune and Sigma. **Table S9**. Dimensions that worsened for patient who worsened overall, comparing Attune and Sigma. **Fig. S1**. Comparison of EQ-5D-3L profiles at baseline and at 11–22 months for Attune and Sigma patients. **Table S10**. The most common EQ-5D-3L profiles observed pre-surgery and at follow up, for Attune patients. **Table S11**. the most common EQ-5D-3L profiles observed pre-surgery and at follow up, for Sigma patients. **Table S12**. Regression coefficients for EQ-VAS with EQ-5D-3L dimensions and levels including dummy for knee replacement system, for all patients. **Table S13**. Regression coefficients for EQ-VAS with EQ-5D-3L dimensions and levels, for Attune patients. **Table S14**. Regression coefficients for EQ-VAS with EQ-5D-3L dimensions and levels, for Sigma patients.

## Data Availability

The data are owned by DePuy Synthes; the authors do not have permission to share.
