# Supplementary material for: EQ-5D-3L and quality of life in total knee arthroplasty (TKA) patients: beyond the index scores
Source: J Patient Rep Outcomes. 2022 Aug 30;6:91. doi: 10.1186/s41687-022-00497-0 (PMC9428084; doi:10.1186/s41687-022-00497-0)
Supplement: Supplementary file 1 — Additional file 1: Table S1. Number of patients by level within EQ-5D-3L dimensions at different observation points (all patients). Table S2. Number of Attune patients by level within EQ-5D-3L dimensions at different observation points. Table S3. number of Sigma patients by level within EQ-5D-3L dimensions at different observation points. Table S4. Changes over time in Levels within dimensions for Attune patients. Table S5. Changes over time in Levels within dimensions for Sigma patients. Table S6. Changes over time in levels within dimensions, comparing Attune and Sigma. Table S7. Classification of Change from pre- to post-surgery, comparing Attune and Sigma. Table S8. Number of worse dimensions for patients whose quality of life worsened, comparing Attune and Sigma. Table S9. Dimensions that worsened for patient who worsened overall, comparing Attune and Sigma. Fig. S1. Comparison of EQ-5D-3L profiles at baseline and at 11–22 months for Attune and Sigma patients. Table S10. The most common EQ-5D-3L profiles observed pre-surgery and at follow up, for Attune patients. Table S11. the most common EQ-5D-3L profiles observed pre-surgery and at follow up, for Sigma patients. Table S12. Regression coefficients for EQ-VAS with EQ-5D-3L dimensions and levels including dummy for knee replacement system, for all patients. Table S13. Regression coefficients for EQ-VAS with EQ-5D-3L dimensions and levels, for Attune patients. Table S14. Regression coefficients for EQ-VAS with EQ-5D-3L dimensions and levels, for Sigma patients. [file 41687_2022_497_MOESM1_ESM.pdf]

EQ-5D-3L and Quality of Life in Total Knee Arthroplasty (TKA) Patients: Beyond the Index Scores

Supplementary tables

Table S1: Number of patients by level within EQ-5D-3L dimensions at different observation points (all patients).

		Pre-surgery		<11 months		11-22 months	
	Level	Number	%	Number	%	Number	%
Mobility	1	261	16.4	1126	71.0	1251	78.8
	2	1323	83.4	461	29.0	336	21.2
	3	3	0.2	0	0.0	0	0.0
Self Care	1	1258	79.3	1456	91.7	1497	94.3
	2	323	20.4	131	8.3	88	5.5
	3	6	0.4	0	0.0	2	0.1
Usual Activities	1	219	13.8	867	54.6	1113	70.1
	2	1245	78.4	690	43.5	461	29.0
	3	123	7.8	30	1.9	13	0.8
Pain & Discomfort	1	26	1.6	470	29.6	888	56.0
	2	1130	71.2	1066	67.2	667	42.0
	3	431	27.2	51	3.2	32	2.0
Anxiety & Depression	1	975	61.4	1254	79.0	1333	84.0
	2	563	35.5	320	20.2	234	14.7
	3	49	3.1	13	0.8	20	1.3

Table S2: Number of Attune patients by level within EQ-5D-3L dimensions at different observation points.

		Pre-surgery		<11 months		11-22 months	
	Level	Number	%	Number	%	Number	%
Mobility	1	114	12.2	636	67.9	714	76.2
	2	820	87.5	301	32.1	223	23.8
	3	3	0.3	0	0.0	0	0.0
Self Care	1	734	78.3	861	91.9	891	95.1
	2	199	21.2	76	8.1	44	4.7
	3	4	0.4	0	0.0	2	0.2
Usual Activities	1	133	14.2	511	54.5	670	71.5
	2	741	79.1	407	43.4	264	28.2
	3	63	6.7	19	2.0	3	0.3
Pain & Discomfort	1	15	1.6	280	29.9	545	58.2
	2	675	72.0	628	67.0	375	40.0
	3	247	26.4	29	3.1	17	1.8
Anxiety & Depression	1	564	60.2	736	78.5	797	85.1
	2	340	36.3	193	20.6	130	13.9
	3	33	3.5	8	0.9	10	1.1

Table S3: number of Sigma patients by level within EQ-5D-3L dimensions at different observation points.

		Pre-surgery		<11 months		11-22 months	
	Level	Number	%	Number	%	Number	%
Mobility	1	147	22.6	490	75.4	537	82.6
	2	503	77.4	160	24.6	113	17.4
	3	0	0.0	0	0.0	0	0.0
Self Care	1	524	80.6	595	91.5	606	93.2
	2	124	19.1	55	8.5	44	6.8
	3	2	0.3	0	0.0	0	0.0
Usual Activities	1	86	13.2	356	54.8	443	68.2
	2	504	77.5	283	43.5	197	30.3
	3	60	9.2	11	1.7	10	1.5
Pain & Discomfort	1	11	1.7	190	29.2	343	52.8
	2	455	70.0	438	67.4	292	44.9
	3	184	28.3	22	3.4	15	2.3
Anxiety & Depression	1	411	63.2	518	79.7	536	82.5
	2	223	34.3	127	19.5	104	16.0
	3	16	2.5	5	0.8	10	1.5

Table S4: Changes over time in Levels within dimensions for Attune patients.

	Change	Pre- to <11 months		Pre- to 11-22 months		<11 months to 11-22 months	
		N	%	N	%	N	%
	1-1	98	86.0	111	97.4	541	85.1
	1-2	*16*	*14.0*	*3*	*2.6*	*95*	*14.9*
	1-3						
	**2-1**	**536**	**65.4**	**601**	**73.3**	**173**	**57.5**
Mobility	2-2	284	34.6	219	26.7	128	42.5
	2-3						
	**3-1**	**2**	**66.7**	**2**	**66.7**		
	**3-2**	**1**	**33.3**	**1**	**33.3**		
	3-3						
	1-1	706	96.2	718	97.8	839	97.4
	1-2	*28*	*3.8*	*15*	*2.0*	*21*	*2.4*
	1-3			*1*	*0.1*	*1*	*0.1*
	**2-1**	**152**	**76.4**	**169**	**84.9**	**52**	**68.4**
Self-Care	2-2	47	23.6	29	14.6	23	30.3
	2-3			*1*	*0.5*	*1*	*1.3*
	3-1	3	75.0	4	100.0		
	**3-2**	**1**	**25.0**				
	**3-3**						
	1-1	100	75.2	118	88.7	440	86.1
	1-2	*33*	*24.8*	*15*	*11.3*	*71*	*13.9*
	1-3						
	**2-1**	**383**	**51.7**	**515**	**69.5**	**219**	**53.8**
Usual Activities	2-2	342	46.2	224	30.2	186	45.7
	2-3	*16*	*2.2*	*2*	*0.3*	*2*	*0.5*
	**3-1**	**28**	**44.4**	**37**	**58.7**	**11**	**57.9**
	**3-2**	**32**	**50.8**	**25**	**39.7**	**7**	**36.8**
	3-3	3	4.8	1	1.6	1	5.3
	1-1	8	53.3	12	80.0	233	83.2
	1-2	*7*	*46.7*	*3*	*20.0*	*46*	*16.4*
	1-3					*1*	*0.4*
	**2-1**	**215**	**31.9**	**415**	**61.5**	**308**	**49.0**
Pain & Discomfort	2-2	449	66.5	257	38.1	309	49.2
	2-3	*11*	*1.6*	*3*	*0.4*	*11*	*1.8*
	**3-1**	**57**	**23.1**	**118**	**47.8**	**4**	**13.8**
	**3-2**	**172**	**69.6**	**115**	**46.6**	**20**	**69.0**
	3-3	18	7.3	14	5.7	5	17.2
	1-1	515	91.3	532	94.3	685	93.1
	1-2	*48*	*8.5*	*31*	*5.5*	*51*	*6.9*
	1-3	*1*	*0.2*	*1*	*0.2*	*0*	*0.0*
	**2-1**	**208**	**61.2**	**247**	**72.6**	**111**	**57.5**
Anxiety & Depression	2-2	126	37.1	88	25.9	74	38.3
	2-3	*6*	*1.8*	*5*	*1.5*	*8*	*4.1*
	**3-1**	**13**	**39.4**	**18**	**54.5**	**1**	**12.5**
	**3-2**	**19**	**57.6**	**11**	**33.3**	**5**	**62.5**
	3-3	1	3.0	4	12.1	2	25.0

Bold = improvements, Italics = worsening, no bold or italic = no change.

Table S5: Changes over time in Levels within dimensions for Sigma patients.

	Change	Pre- to <11 months		Pre- to 11-22 months		<11 months to 11-22 months	
		N	%	N	%	N	%
Mobility	1-1	137	93.2	140	95.2	438	89.4
	1-2	*10*	*6.8*	*7*	*4.8*	*52*	*10.6*
	1-3						
	**2-1**	**353**	**70.2**	**397**	**78.9**	**99**	**61.9**
	2-2	150	29.8	106	21.1	61	38.1
	2-3						
	**3-1**						
	**3-2**						
	3-3						
Self-Care	1-1	499	95.2	504	96.2	570	95.8
	1-2	*25*	*4.8*	*20*	*3.8*	*25*	*4.2*
	1-3						
	**2-1**	**94**	**75.8**	**100**	**80.6**	**36**	**65.5**
	2-2	30	24.2	24	19.4	19	34.5
	2-3						
	**3-1**	**2**	**100.0**	**2**	**100.0**		
	**3-2**						
	3-3						
Usual Activities	1-1	71	82.6	78	90.7	301	84.6
	1-2	*15*	*17.4*	*8*	*9.3*	*53*	*14.9*
	1-3					*2*	*0.6*
	**2-1**	**264**	**52.4**	**338**	**67.1**	**139**	**49.1**
	2-2	233	46.2	158	31.3	136	48.1
	2-3	*7*	*1.4*	*8*	*1.6*	*8*	*2.8*
	**3-1**	**21**	**35.0**	**27**	**45.0**	**3**	**27.3**
	**3-2**	**35**	**58.3**	**31**	**51.7**	**8**	**72.7**
	3-3	4	6.7	2	3.3		0.0
Pain & Discomfort	1-1	6	54.5	7	63.6	141	74.2
	1-2	*5*	*45.5*	*4*	*36.4*	*49*	*25.8*
	1-3						
	**2-1**	**144**	**31.6**	**259**	**56.9**	**197**	**45.0**
	2-2	300	65.9	188	41.3	230	52.5
	2-3	*11*	*2.4*	*8*	*1.8*	*11*	*2.5*
	**3-1**	**40**	**21.7**	**77**	**41.8**	**5**	**22.7**
	**3-2**	**133**	**72.3**	**100**	**54.3**	**13**	**59.1**
	3-3	11	6.0	7	3.8	4	18.2
Anxiety & Depression	1-1	377	91.7	383	93.2	475	91.7
	1-2	*34*	*8.3*	*28*	*6.8*	*39*	*7.5*
	1-3					*4*	*0.8*
	**2-1**	**134**	**60.1**	**146**	**65.5**	**60**	**47.2**
	2-2	86	38.6	69	30.9	63	49.6
	2-3	*3*	*1.3*	*8*	*3.6*	*4*	*3.1*
	**3-1**	**7**	**43.8**	**7**	**43.8**	**1**	**20.0**
	**3-2**	**7**	**43.8**	**7**	**43.8**	**2**	**40.0**
	3-3	2	12.5	2	12.5	2	40.0

Bold = improvements, Italics = worsening, no bold or italic = no change.

Table S6: Changes over time in levels within dimensions, comparing Attune and Sigma.

	Change	Pre- to <11 months (%)		Pre- to 11-22 months (%)		<11 months to 11-22 months (%)	
		Attune	Sigma	Attune	Sigma	Attune	Sigma
Mobility	1-1	86.0	93.2	97.4	95.2	85.1	89.4
	1-2	14.0	6.8	2.6	4.8	14.9	10.6
	1-3						
	2-1	65.4	70.2	73.3	78.9	57.5	61.9
	2-2	34.6	29.8	26.7	21.1	42.5	38.1
	2-3						
	3-1	66.7		66.7			
	3-2	33.3		33.3			
	3-3						
Self-Care	1-1	96.2	95.2	97.8	96.2	97.4	95.8
	1-2	3.8	4.8	2.0	3.8	2.4	4.2
	1-3			0.1		0.1	
	2-1	76.4	75.8	84.9	80.6	68.4	65.5
	2-2	23.6	24.2	14.6	19.4	30.3	34.5
	2-3			0.5		1.3	
	3-1	75.0	100.0	100.0	100.0		
	3-2	25.0					
	3-3						
Usual Activities	1-1	75.2	82.6	88.7	90.7	86.1	84.6
	1-2	24.8	17.4	11.3	9.3	13.9	14.9
	1-3						0.6
	2-1	51.7	52.4	69.5	67.1	53.8	49.1
	2-2	46.2	46.2	30.2	31.3	45.7	48.1
	2-3	2.2	1.4	0.3	1.6	0.5	2.8
	3-1	44.4	35.0	58.7	45.0	57.9	27.3
	3-2	50.8	58.3	39.7	51.7	36.8	72.7
	3-3	4.8	6.7	1.6	3.3	5.3	0.0
Pain & Discomfort	1-1	53.3	54.5	80.0	63.6	83.2	74.2
	1-2	46.7	45.5	20.0	36.4	16.4	25.8
	1-3					0.4	
	2-1	31.9	31.6	61.5	56.9	49.0	45.0
	2-2	66.5	65.9	38.1	41.3	49.2	52.5
	2-3	1.6	2.4	0.4	1.8	1.8	2.5
	3-1	23.1	21.7	47.8	41.8	13.8	22.7
	3-2	69.6	72.3	46.6	54.3	69.0	59.1
	3-3	7.3	6.0	5.7	3.8	17.2	18.2
Anxiety & Depression	1-1	91.3	91.7	94.3	93.2	93.1	91.7
	1-2	8.5	8.3	5.5	6.8	6.9	7.5
	1-3	0.2		0.2		0.0	0.8
	2-1	61.2	60.1	72.6	65.5	57.5	47.2
	2-2	37.1	38.6	25.9	30.9	38.3	49.6
	2-3	1.8	1.3	1.5	3.6	4.1	3.1
	3-1	39.4	43.8	54.5	43.8	12.5	20.0
	3-2	57.6	43.8	33.3	43.8	62.5	40.0
	3-3	3.0	12.5	12.1	12.5	25.0	40.0

Table S7: Classification of Change from pre- to post-surgery, comparing Attune and Sigma.

	Attune				Sigma			
	< 11 months		11-22 months		< 11 months		11-22 months	
	N	%	N	%	N	%	N	%
Improve	816	76.5	833	86.1	534	73.9	552	83.8
Worsen	83	7.8	30	3.1	51	7.1	27	4.1
No change	90	8.4	60	6.2	75	10.4	36	5.5
Mixed	77	7.2	45	4.6	63	8.7	44	6.7
Total	1066		968		723		659	

Table S8: Number of worse dimensions for patients whose quality of life worsened, comparing Attune and Sigma

Number of worse dimensions	Attune				Sigma			
	< 11 months		11-22 months		< 11 months		11-22 months	
	N	%	N	%	N	%	N	%
1	62	74.7	24	80.0	39	76.5	16	59.3
2	13	15.7	5	16.7	9	17.6	5	18.5
3	7	8.4	1	3.3	2	3.9	4	14.8
4	1	1.2			1	2.0	2	7.4

Table S9: Dimensions that worsened for patient who worsened overall, comparing Attune and Sigma.

EQ-5D Dimension	Attune				Sigma			
	< 11 months		11-22 months		< 11 months		11-22 months	
	N	%	N	%	N	%	N	%
Mobility	10	12.0	2	6.7	6	11.8	5	18.5
Self-Care	19	22.9	11	36.7	14	27.5	11	40.7
Usual Activities	33	39.8	10	33.3	14	27.5	12	44.4
Pain & Discomfort	12	14.5	3	10.0	14	27.5	7	25.9
Anxiety & Depression	39	47.0	11	36.7	19	37.3	11	40.7

Percentages are of number of patients who worsened.

Figure S1: Comparison of EQ-5D-3L profiles at baseline and at 11-22 months for Attune and Sigma patients.

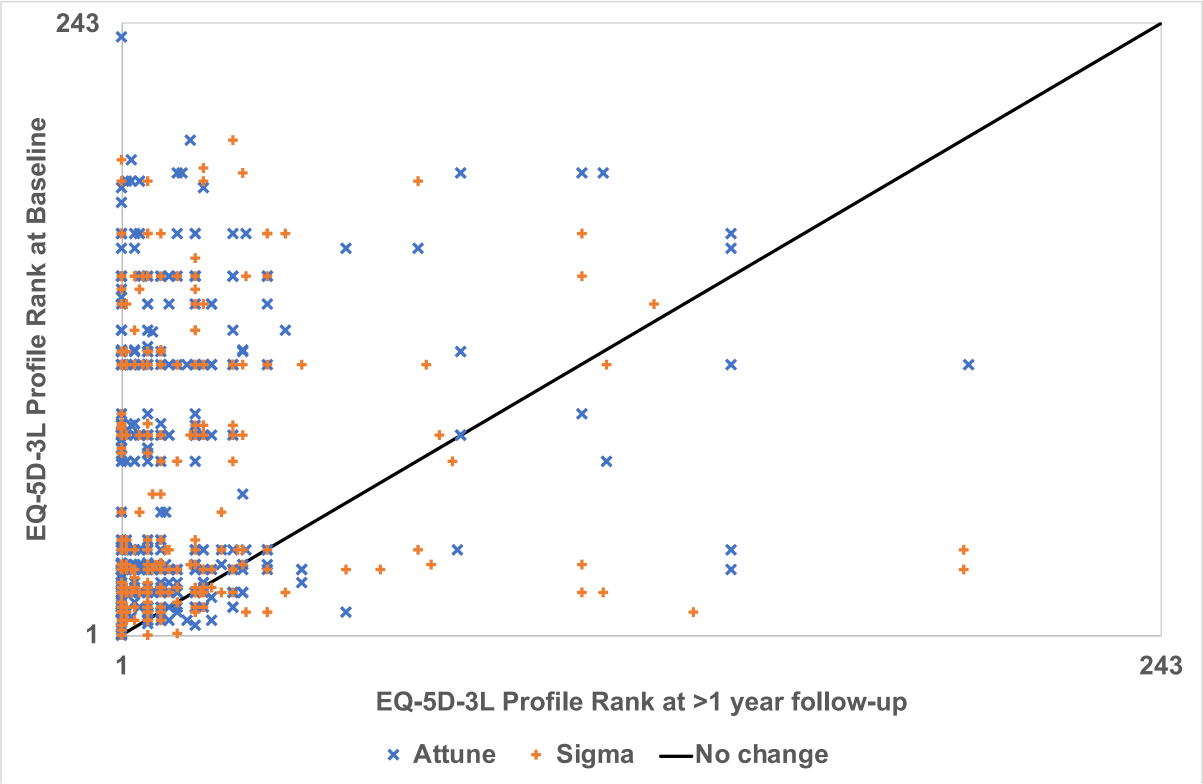

Table S10: The most common EQ-5D-3L profiles observed pre-surgery and at follow up, for Attune patients.

Pre-surgery				<11 months				11-22 months			
Profile	N	%	Cu%	Profile	N	%	Cu%	Profile	N	%	Cu%
21221	249	26.6	26.6	11111	204	21.8	21.8	11111	436	46.5	46.5
21222	127	13.6	40.1	11121	196	20.9	42.7	11121	125	13.3	59.9
21231	68	7.3	47.4	21221	100	10.7	53.4	21221	81	8.6	68.5
22221	52	5.5	52.9	11221	98	10.5	63.8	11221	43	4.6	73.1
21232	49	5.2	58.2	21222	54	5.8	69.6	11211	31	3.3	76.4
22222	49	5.2	63.4	11211	33	3.5	73.1	11112	27	2.9	79.3
21121	48	5.1	68.5	11222	32	3.4	76.5	21222	23	2.5	81.8
11121	39	4.2	72.7	21121	31	3.3	79.8	11122	20	2.1	83.9
11221	39	4.2	76.8	11122	29	3.1	82.9	21121	20	2.1	86.0
22232	35	3.7	80.6	22222	24	2.6	85.5	21111	17	1.8	87.8
22231	21	2.2	82.8	22221	22	2.3	87.8	21211	15	1.6	89.4
21122	19	2.0	84.8	11112	20	2.1	90.0	11222	14	1.5	90.9
22332	13	1.4	86.2								
21332	10	1.1	87.3								
21223	9	1.0	88.3								
21321	9	1.0	89.2								
11222	8	0.9	90.1								

Table S11: the most common EQ-5D-3L profiles observed pre-surgery and at follow up, for Sigma patients.

Pre-surgery				<11 months				11-22 months			
Profile	N	%	Cu%	Profile	N	%	Cu%	Profile	N	%	Cu%
21221	160	24.6	24.6	11121	149	22.9	22.9	11111	280	43.1	43.1
11221	61	9.4	34.0	11111	146	22.5	45.4	11121	109	16.8	59.8
21222	61	9.4	43.4	11221	101	15.5	60.9	11221	45	6.9	66.8
21231	50	7.7	51.1	21221	47	7.2	68.2	21221	32	4.9	71.7
21232	43	6.6	57.7	21222	28	4.3	72.5	11211	29	4.5	76.2
11121	42	6.5	64.2	11122	24	3.7	76.2	11222	22	3.4	79.5
22221	25	3.8	68.0	11222	21	3.2	79.4	21222	17	2.6	82.2
22222	25	3.8	71.8	22222	16	2.5	81.8	11122	16	2.5	84.6
22232	23	3.5	75.4	22221	15	2.3	84.2	22222	14	2.2	86.8
21121	21	3.2	78.6	21121	15	2.3	86.5	11112	13	2.0	88.8
11222	19	2.9	81.5	11211	15	2.3	88.8	21211	7	1.1	89.8
22332	18	2.8	84.3	11112	9	1.4	90.2	21121	6	0.9	90.8
22231	15	2.3	86.6								
21321	8	1.2	87.8								
11122	7	1.1	88.9								
21332	7	1.1	90.0								

Table S12: Regression coefficients for EQ-VAS with EQ-5D-3L dimensions and levels including dummy for knee replacement system, for all patients.

	Pre-surgery		Post-surgery	
	Estimate	Std. Error	Estimate	Std. Error
(Intercept)	91.7	3.10	88.20	0.479
Knee system	-0.485	0.791	-0.762	0.623
Mobility 2	-4.41	1.15	-6.02	0.934
Mobility 3	-8.35	9.99	NA	NA
Selfcare 2	-4.39	1.01	-5.46	1.43
Selfcare 3	2.46	6.53	3.50	8.78
Usual Activity 2	-4.10	1.21	-5.82	0.854
Usual Activity 3	-9.39	1.89	-20.1	3.28
Pain & Discomfort 2	-5.82	3.11	-2.56	0.715
Pain & Discomfort 3	-8.92	3.22	-10.9	2.39
Anxiety & Depression 2	-7.12	0.845	-8.01	0.905
Anxiety & Depression 3	-14.9	2.26	-16.1	2.74
Adjusted R^2	0.171		0.335	

Highlight = Not significantly different from zero at 0.05 level, or cannot be computed (NA)

Table S13: Regression coefficients for EQ-VAS with EQ-5D-3L dimensions and levels, for Attune patients.

	Pre-surgery		Post-surgery	
	Estimate	Std. Error	Estimate	Std. Error
(Intercept)	91.1	4.11	87.8	0.523
Mobility 2	-5.28	1.65	-4.51	1.17
Mobility 3	-12.6	10.6	NA	NA
Selfcare 2	-2.79	1.29	-6.56	1.95
Selfcare 3	9.35	8.17	2.62	9.05
Usual Activity 2	-4.40	1.54	-5.24	1.13
Usual Activity 3	-11.5	2.51	-22.1	5.92
Pain & Discomfort 2	-5.06	4.12	-3.09	0.935
Pain & Discomfort 3	-7.78	4.25	-10.4	3.20
Anxiety & Depression 2	-6.13	1.09	-7.10	1.16
Anxiety & Depression 3	-16.1	2.74	-17.3	3.70
Adjusted R^2	0.135		0.287	

Highlight = Not significantly different from zero at 0.05 level, or cannot be computed (NA)

Table S14: Regression coefficients for EQ-VAS with EQ-5D-3L dimensions and levels, for Sigma patients.

	Pre-surgery		Post-surgery	
	Estimate	Std. Error	Estimate	Std. Error
(Intercept)	91.7	4.65	87.9	0.679
Mobility 2	-3.32	1.61	-9.15	1.57
Mobility 3	NA	NA	NA	NA
Selfcare 2	-7.23	1.63	-3.84	2.11
Selfcare 3	-9.26	10. 9	NA	NA
Usual Activity 2	-3.69	1.96	-6.43	1.31
Usual Activity 3	-6.20	2.89	-18.2	4.11
Pain & Discomfort 2	-6.41	4.75	-2.07	1.11
Pain & Discomfort 3	-9.89	4.94	-12.4	3.63
Anxiety & Depression 2	-8.57	1.34	-8.82	1.45
Anxiety & Depression 3	-21.8	3.85	-13.9	4.12
Adjusted R^2	0.227		0.394	

Highlight = Not significantly different from zero at 0.05 level, or cannot be computed (NA)
